# Supplementary material for: Lipid dependence of connexin-32 gap junction channel conformations
Source: Nat Commun. 2025 Dec 5;17:316. doi: 10.1038/s41467-025-67004-z (PMC12789061; doi:10.1038/s41467-025-67004-z)
Supplement: Supplementary file 1 — Supplementary Information [file 41467_2025_67004_MOESM1_ESM.pdf]

**Supplementary Information**

**Title:** Lipid dependence of connexin-32 gap junction channel conformations

**Authors:** Pia Lavriha[1,2], Carina Fluri[2], Jorge Enrique Hernández González [3*]

and Volodymyr M. Korkhov[1,2*]

**Affiliations:**

[1] Laboratory of Biomolecular Research, Paul Scherrer Institute, Villigen, Switzerland

[2] Institute of Molecular Biology and Biophysics, ETH Zurich, Switzerland

[3] Department of Physics, Institute for Biosciences, Letters and Exact Sciences, Sao Paulo State University, São José do Rio Preto, Brazil

[*] Corresponding author: jorge.hernandez@unesp.br, volodymyr.korkhov@psi.ch

## Supplementary Figures

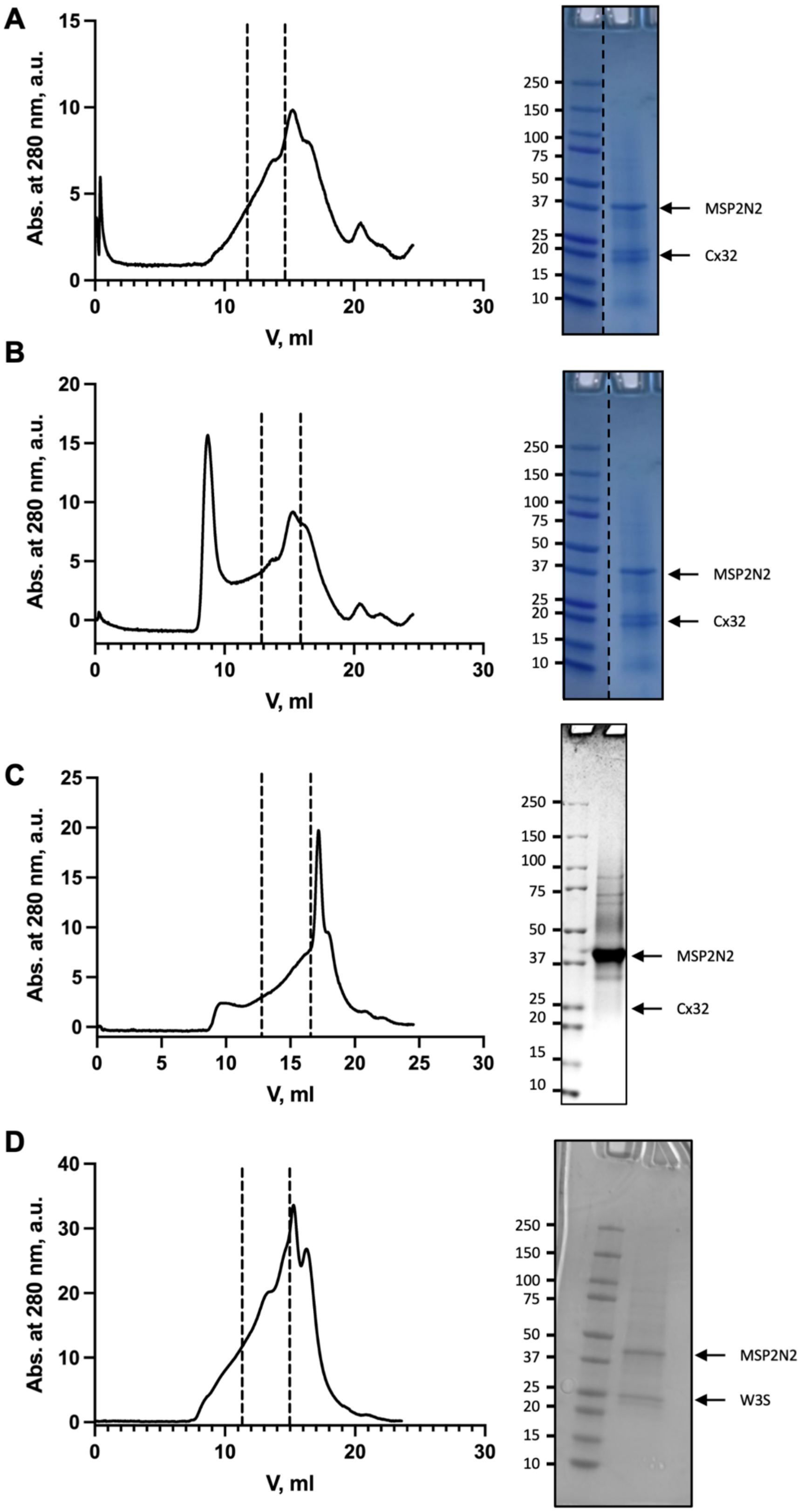

**Figure S1. Size exclusion chromatograms and SDS PAGE gels of nanodisc reconstitution** of **(A)** Cx32 in POPC, **(B)** Cx32 in LPL, **(C)** W3S in POPC, and **(D)** Cx32, purified in the absence of CHS, in POPC nanodisc, using MSP2N2 as the nanodisc scaffold protein.

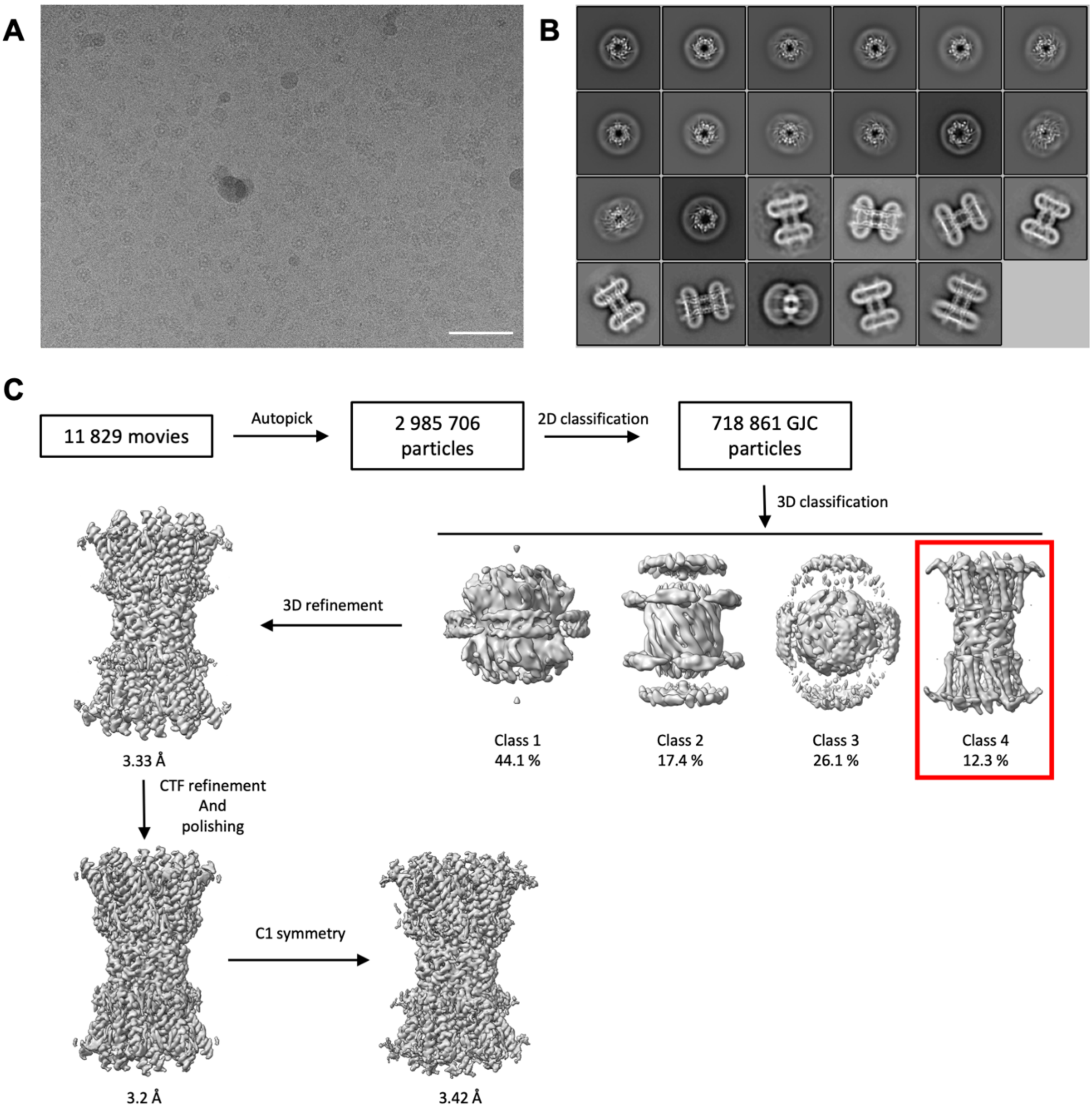


**Figure S2. Cryo-EM structure determination of Cx32 GJC in POPC nanodisc. (A)** Cryo-EM micrograph of Cx32, reconstituted in POPC-containing nanodisc. Scale bar = 50 nm. **(B)** 2D classes of Cx32 GJC particles, used for 3D classification. **(C)** Cryo-EM data processing pipeline for Cx32 GJC in POPC nanodisc.

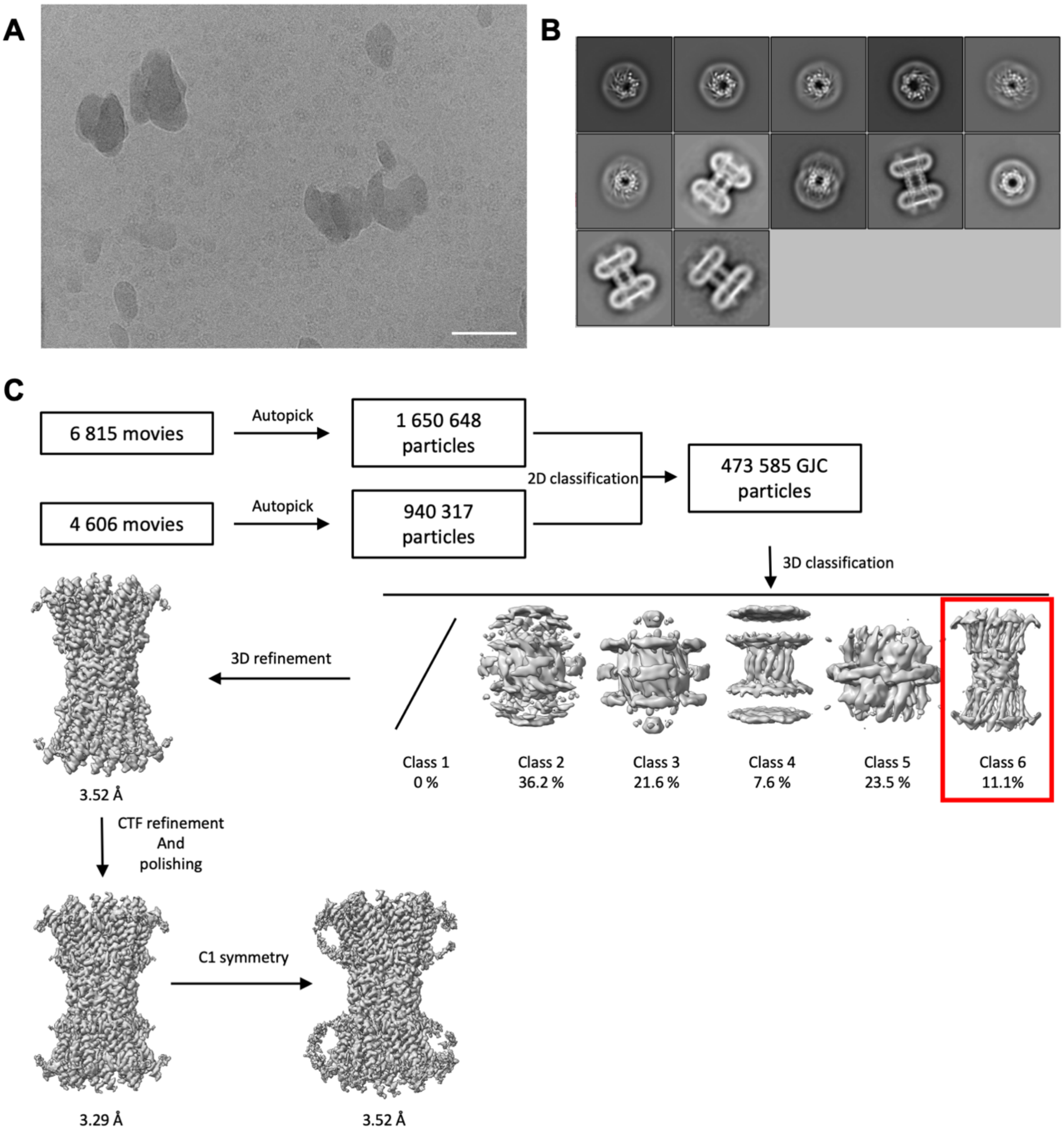


**Figure S3. Cryo-EM structure determination of Cx32 GJC in LPL nanodisc. (A)** Cryo-EM micrograph of Cx32, reconstituted in LPL-containing nanodisc. Scale bar = 50 nm. **(B)** 2D classes of Cx32 GJC particles, used for 3D classification. **(C)** Cryo-EM data processing pipeline for Cx32 GJC in LPL nanodisc.

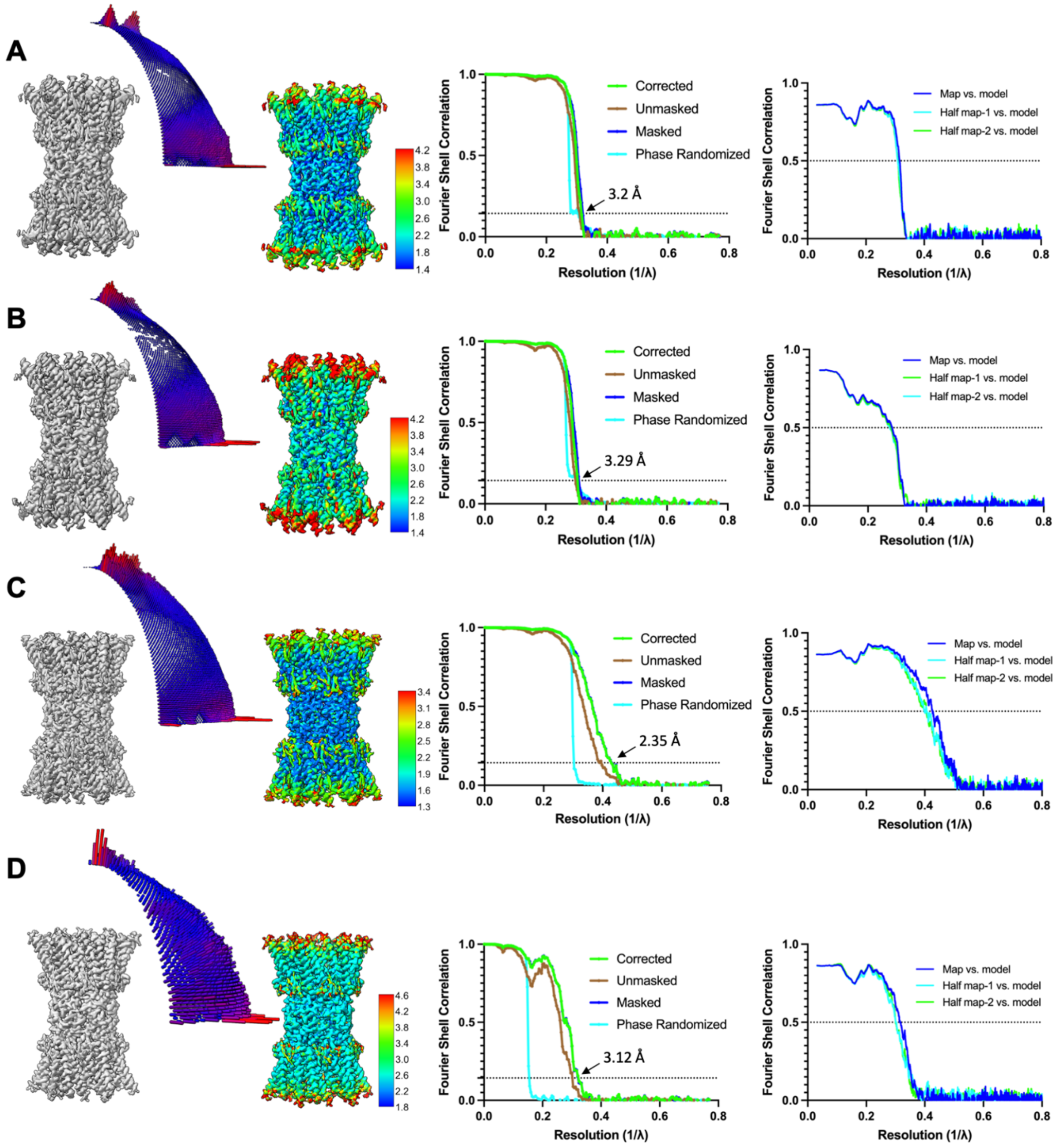


**Figure S4. Angular distribution, local resolution, and Fourier Shell Correlation (FSC) curves.** **(A)** Cx32 GJC in POPC nanodisc. **(B)** Cx32 GJC in LPL nanodisc. **(C)** W3S GJC in POPC nanodisc. **(D)** Cx32 GJC, without CHS, in POPC nanodisc.

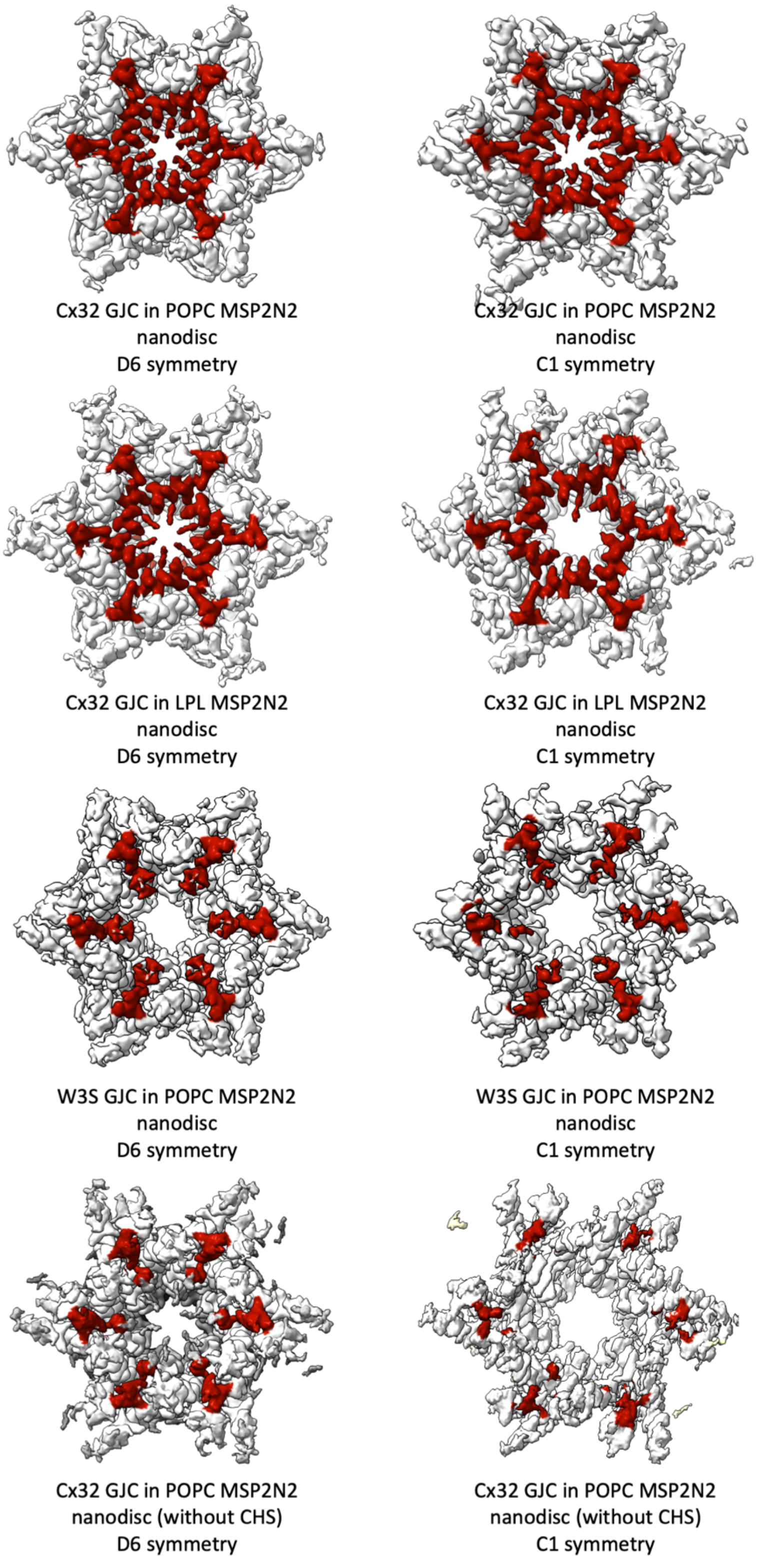


**Figure S5. Top view comparison of Cx32 GJC nanodisc maps in D6 and C1 symmetry.** The densities corresponding to the N-terminus are represented in red.

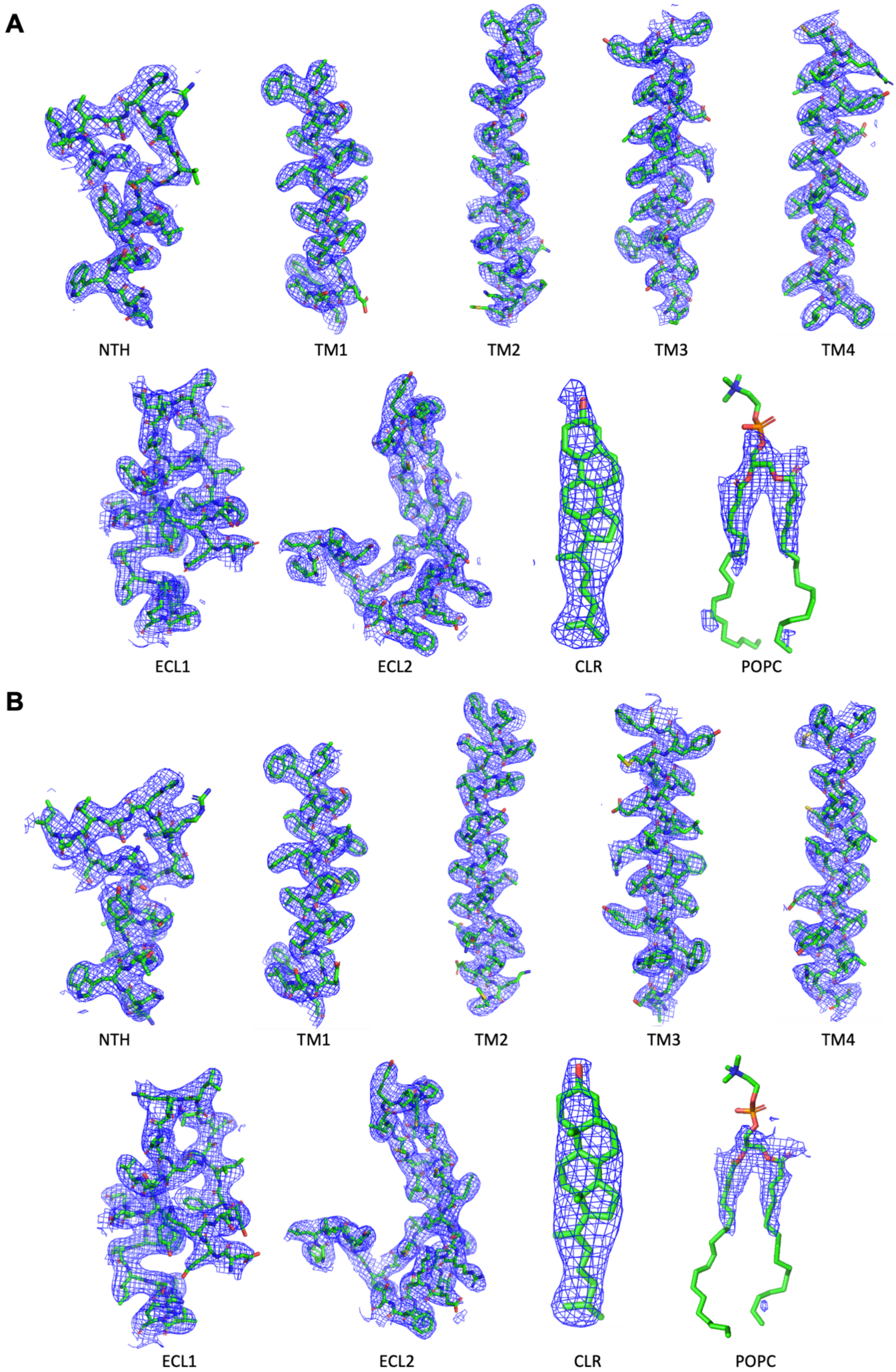


**Figure S6. Cryo-EM density map features of Cx32 GJC in (A) POPC-containing and (B) LPL-containing nanodiscs.**

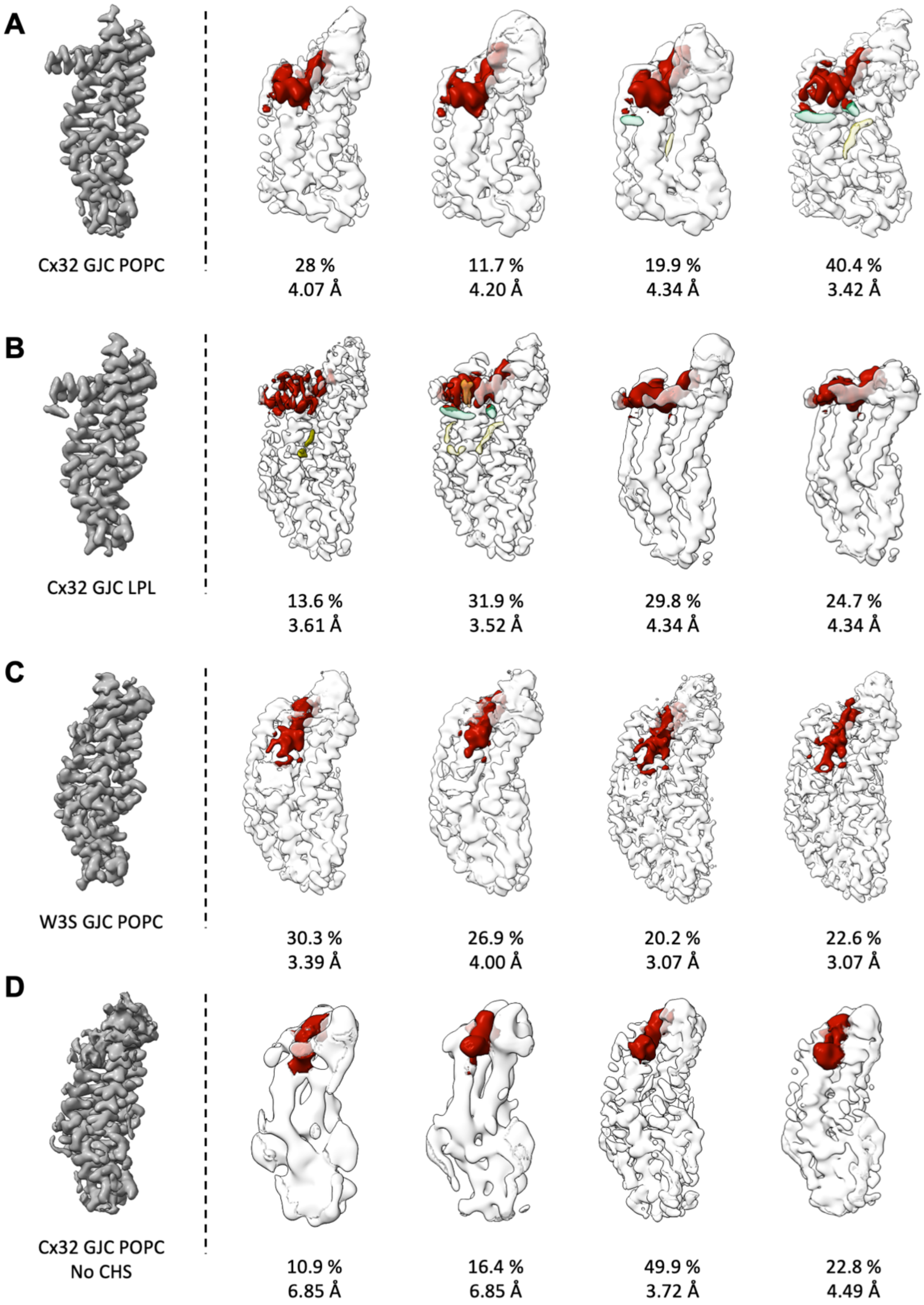


**Figure S7. Protomer-focused classification. (A)** Cx32 GJC in POPC nanodisc. **(B)** Cx32 GJC in LPL nanodisc. **(C)** W3S GJC in nanodisc. **(D)** Cx32 GJC, without CHS, in nanodisc. Red coloured density corresponds to the NTH.

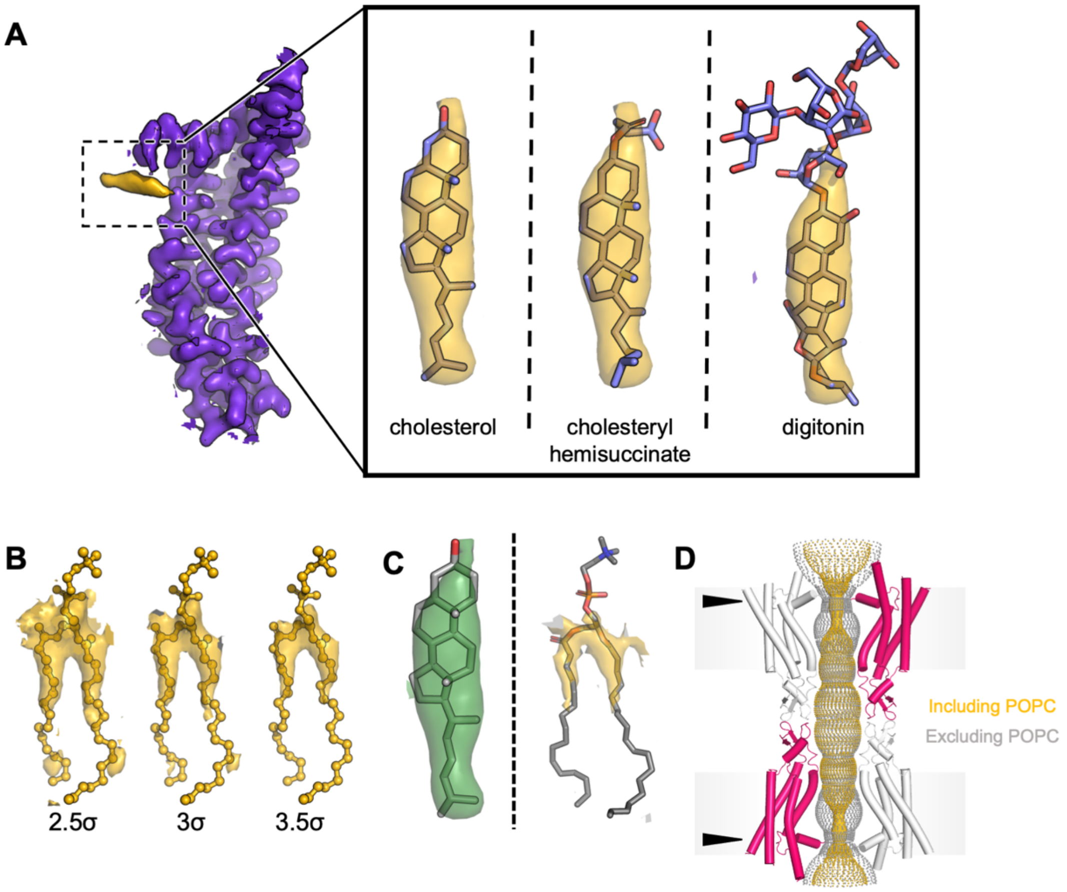


**Figure S8. Effect of lipid-2 on Cx32 GJC NTH. (A)** Lipid-2 density can accommodate cholesterol, or cholesteryl hemisuccinate (CHS), and digitonin, used during protein purification. **(B)** Cx32 GJC reconstituted in LPL-containing nanodisc has the similar densities as Cx32 GJC, reconstituted in POPC containing nanodisc. **(C)** Densities of lipid-2 and lipid-3 in the cryo-EM map of the Cx32 GJC in LPL-containing nanodiscs. **(D)** HOLE analysis of the pore conduction pathway of Cx32 GJC in LPL-containing nanodiscs. The pathway colored in yellow includes POPC in the calculation, whereas the grey excludes it. The arrows represent the points of pore constriction due to NTH rearrangement.

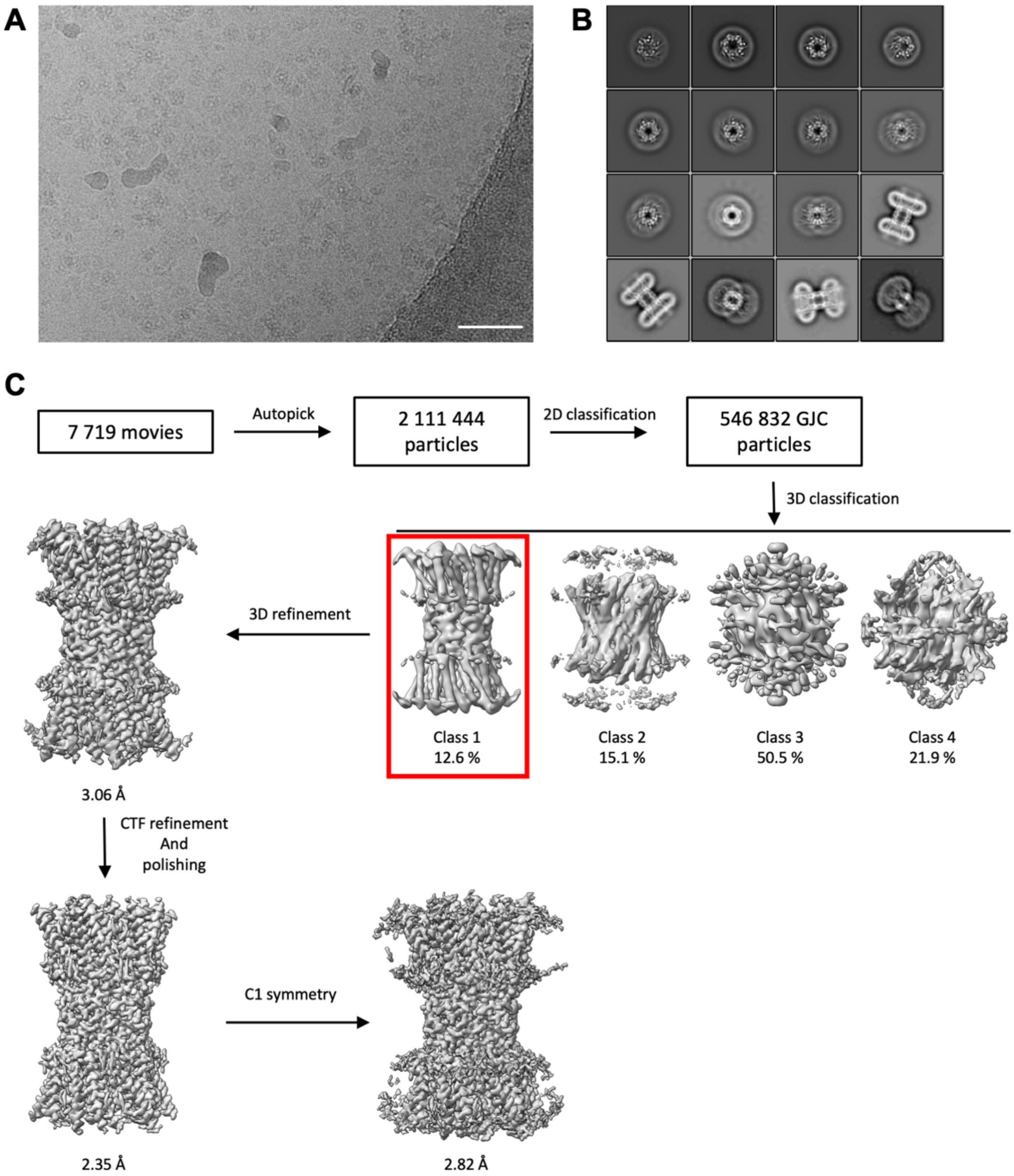


**Figure S9. Cryo-EM structure determination of W3S GJC in nanodisc. (A)** Cryo-EM micrograph of W3S, reconstituted in nanodisc. Scale bar = 50 nm. **(B)** 2D classes of Cx32 GJC particles, used for 3D classification. **(C)** Cryo-EM data processing pipeline for W3S GJC in nanodisc.

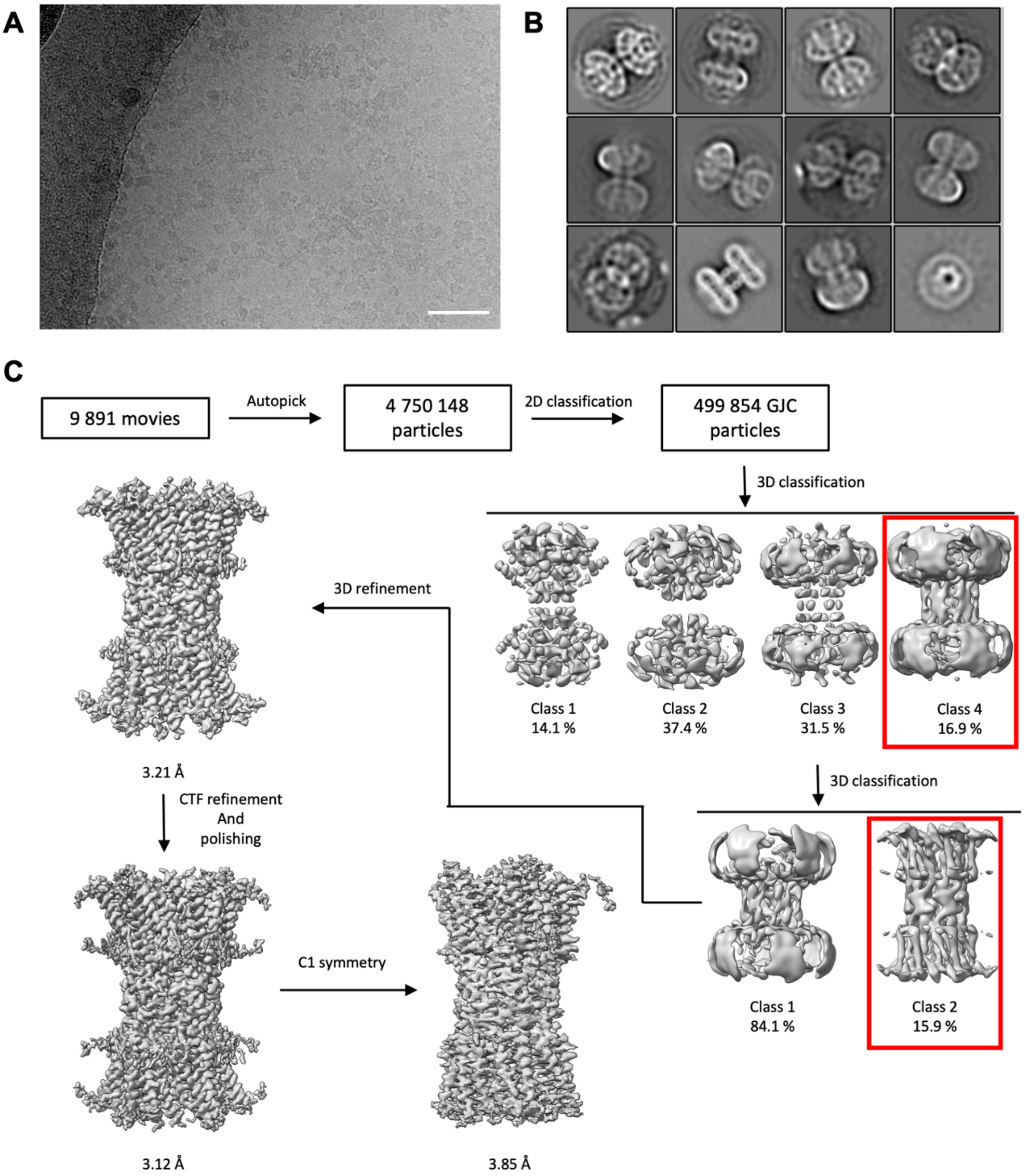


**Figure S10. Cryo-EM structure determination of Cx32 GJC, purified in the absence of CHS, in nanodisc. (A)** Cryo-EM micrograph of Cx32, purified without CHS, in nanodisc. Scale bar = 50 nm. **(B)** 2D classes of Cx32 GJC particles, used for 3D classification. **(C)** Cryo-EM data processing pipeline for Cx32 GJC, purified without CHS, in nanodisc.

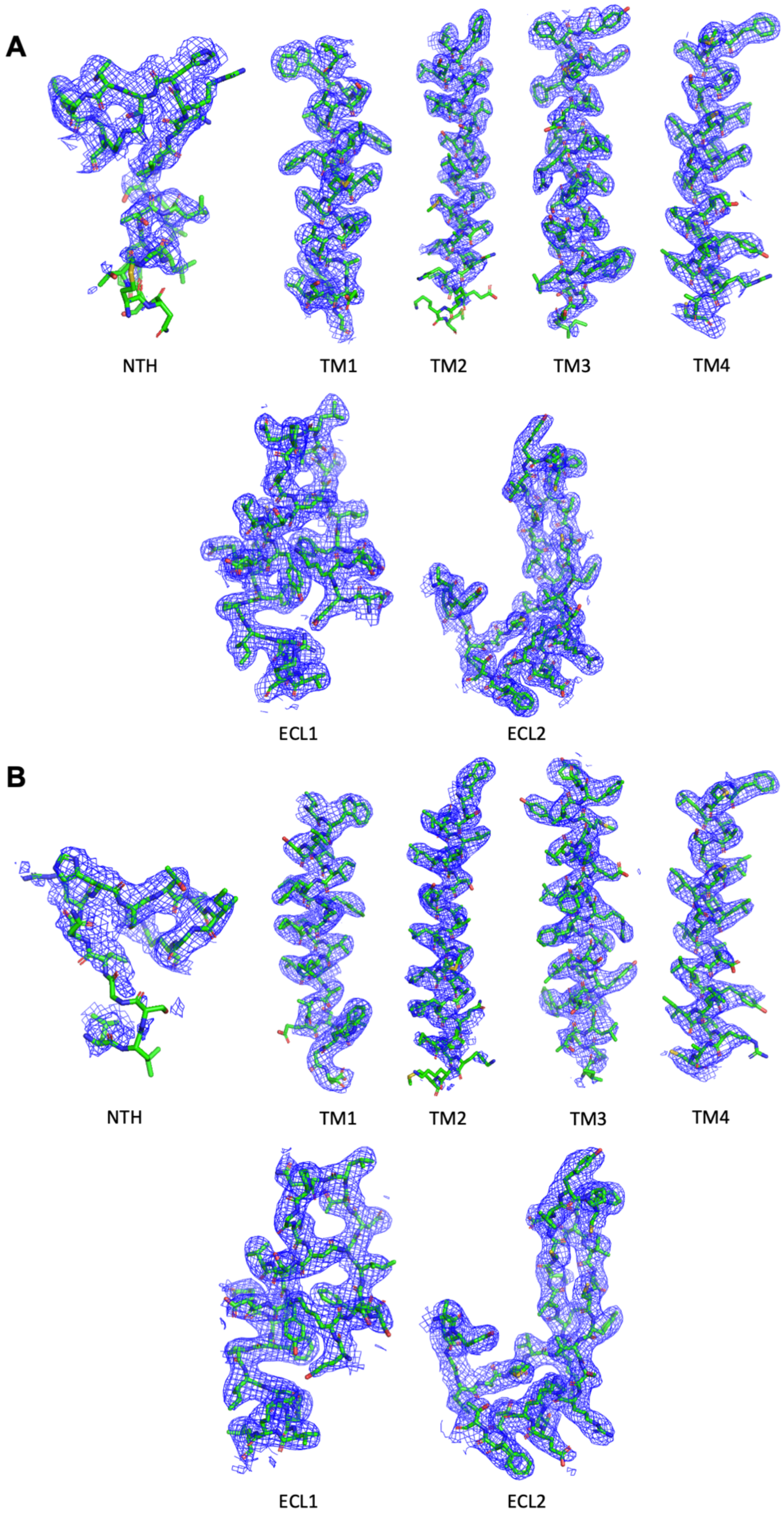


**Figure S11. Cryo-EM density map features of (A) W3S GJC and (B) Cx32 GJC without CHS, in nanodisc.**

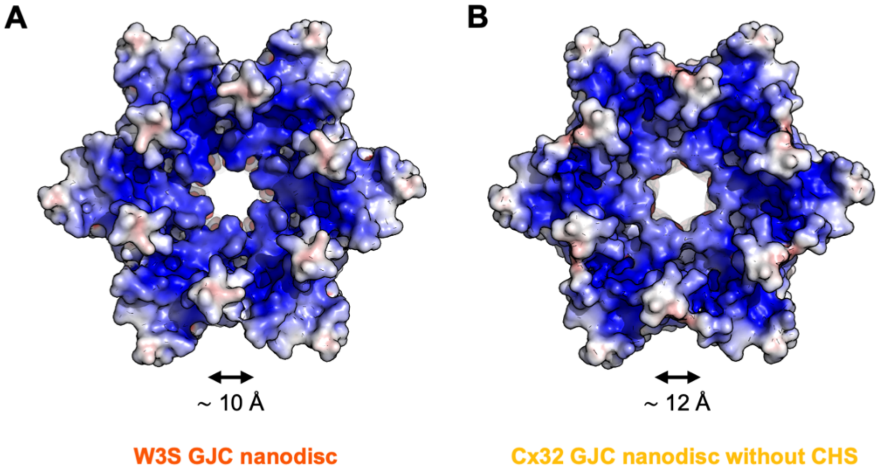


**Figure S12. Electrostatic surface potential of (A) W3S GJC and (B) Cx32 GJC, without CHS, in nanodisc.**

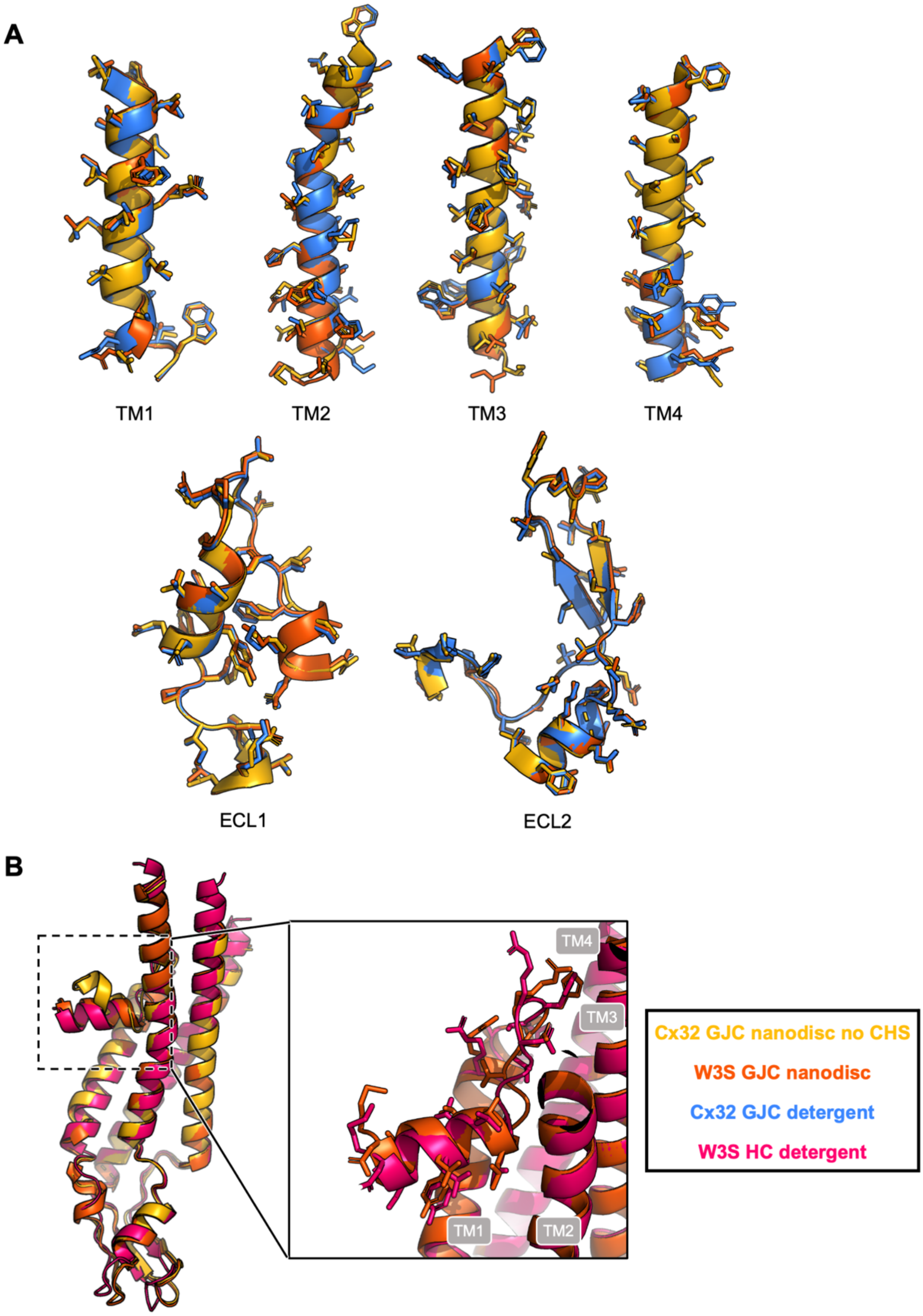


**Figure S13. Comparison of W3S and Cx32, without CHS, GJC structures in detergent and nanodisc. (A)** Comparison of TMH and ECL of W3S and Cx32, without CHS, GJC in nanodiscs

compared to Cx32 GJC structure in detergent. **(B)** Comparison of W3S GJC in nanodisc to W3S HC in detergent.

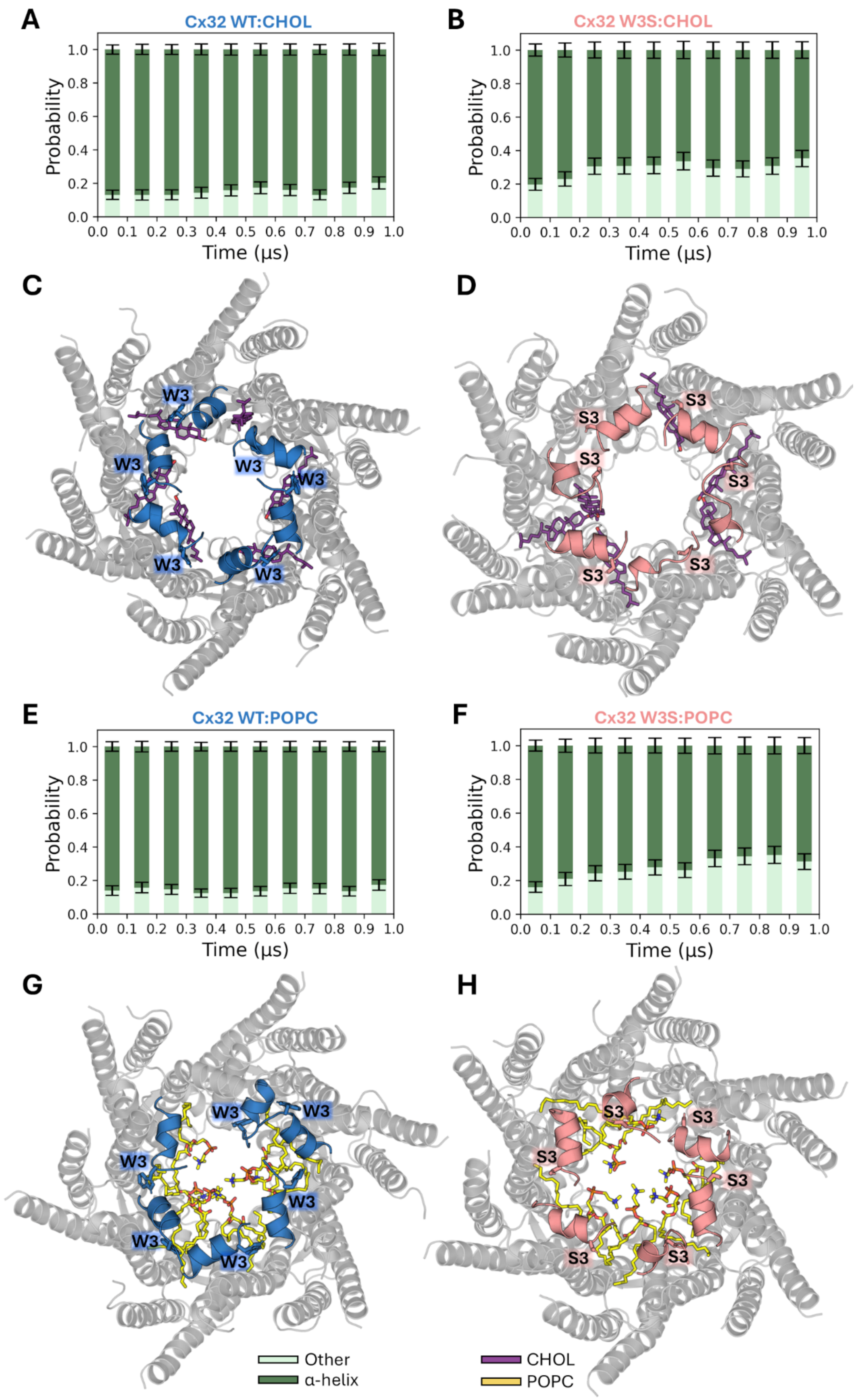


**Figure S14. Time evolution of the secondary structure of NTH residues in Cx32 bound to either CHOL or POPC during 1 µs MD simulations. (A-B), (E-F)** represent the mean probabilities of the

NTH residues 3-5 and 9-11 adopting an α-helical or another type of conformation (mainly turn and random coil) for the indicated systems throughout the simulation time. For each system, the plotted mean probabilities were calculated by averaging the per-residue probabilities for the selected residues in every protein chain and over the two replicate MD simulations (n=12) in 0.1 μs intervals (i.e., 0-0.1, ..., 0.9-1 μs). Other types of secondary structure are omitted. **(C-D)**, **(G-H)** show a structural representation of the last frame ($t$=1 μs) in one of the two replicate trajectories for each system. The N-terminal residues 1-11 are colored in blue and salmon in each panel. Statistical analysis is presented in Table S1.

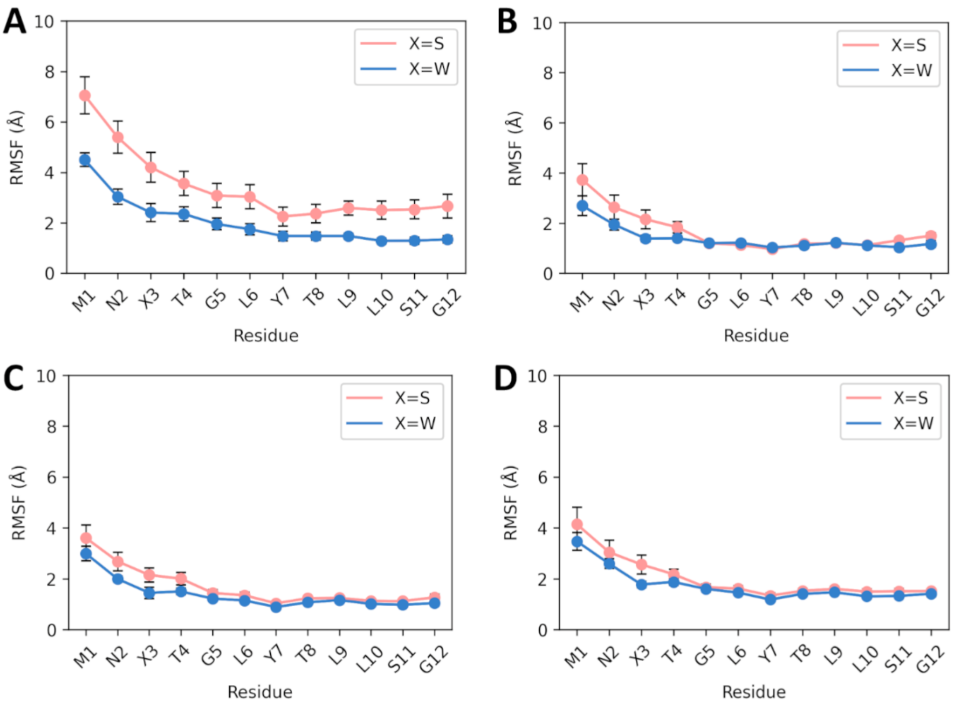


**Figure S15. Root-mean-square fluctuation (RMSF) values for the N-terminal residues of Cx32 wt and Cx32 W3S in the presence and absence of lipids in the pore. (A)** Cx32 wt and Cx32 W3S, (**B**) Cx32 wt:POPC:CHOL and Cx32 W3S:POPC:CHOL, (**C**) Cx32 wt:CHOL and Cx32 W3S:CHOL, and (**D**) Cx32 wt:POPC and Cx32 W3S:POPC. Average RMSF values were calculated for residues 1-12 in the six chains of each protein and during two replicate 1 μs MD simulations for each system (n=12). The first 100 ns of each MD trajectory were excluded from the RMSF analysis. All fluctuations were calculated with respect to the energy-minimized structure of each system and considering the heavy atoms of the selected residues.

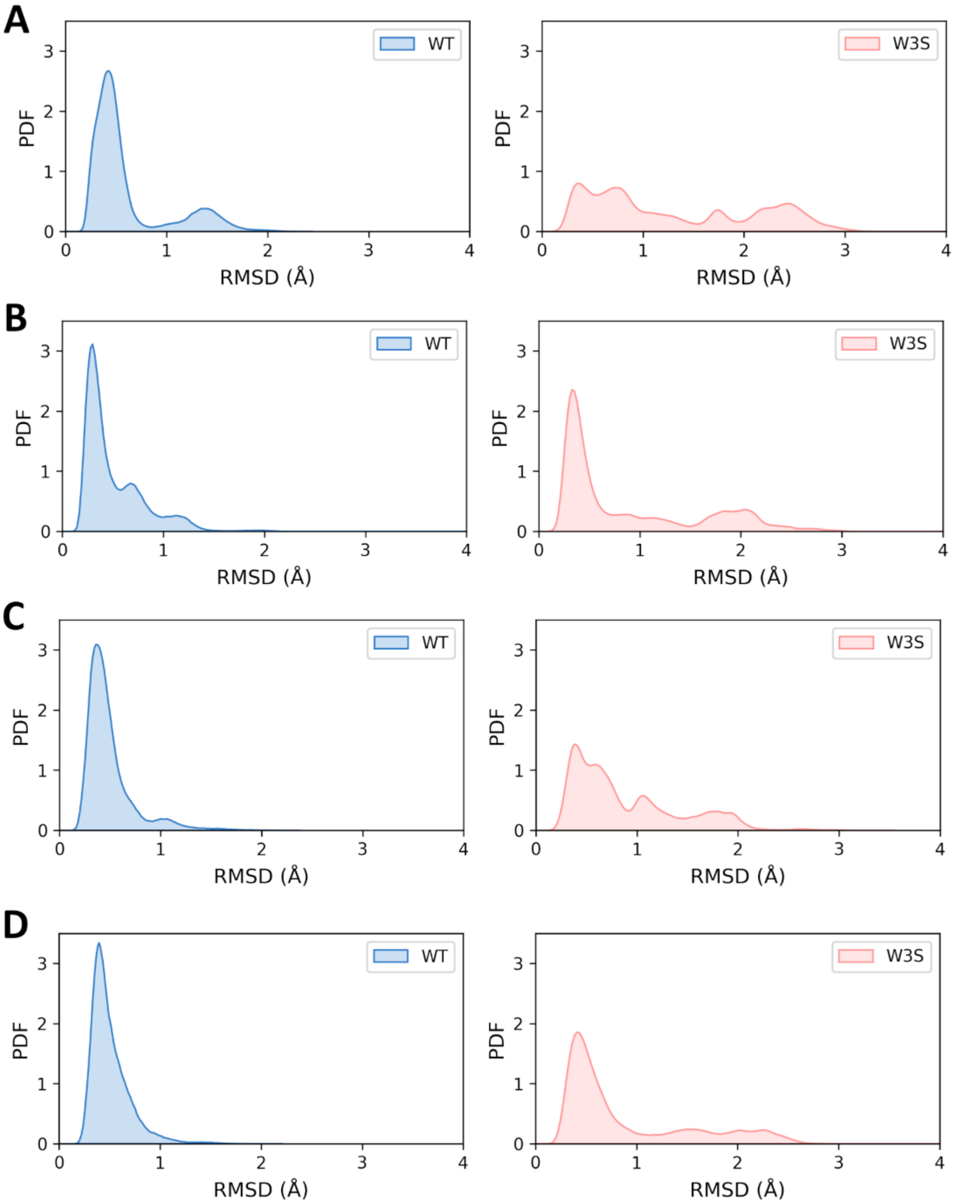


**Figure S16. Distributions of root-mean-square deviation (RMSD) values for the N-terminal residues 3-11 of Cx32 wt and Cx32 W3S in the presence and absence of lipids in the pore. (A)** Cx32 wt and Cx32 W3S, **(B)** Cx32 wt:POPC:CHOL and Cx32 W3S:POPC:CHOL, **(C)** Cx32 wt:CHOL and Cx32 W3S:CHOL, and **(D)** Cx32 wt:POPC and Cx32 W3S:POPC. Average RMSD values were calculated for the backbone of residues 1-10 in the six chains of each protein and during two replicate 1 µs MD simulations for each system (n=12). PDF on the *y* axis stands for probability density function. RMSD values for each N-terminal segment (residues 1-10) were calculated relative to the position of the corresponding segment in the energy-minimized structure of each system, with trajectories fitted onto the initial backbone positions of these residues and after discarding the first 100 ns. Thus, these RMSD calculations focus on the internal motion of the N-terminal residues without reflecting their movement relative to the rest of the protein.

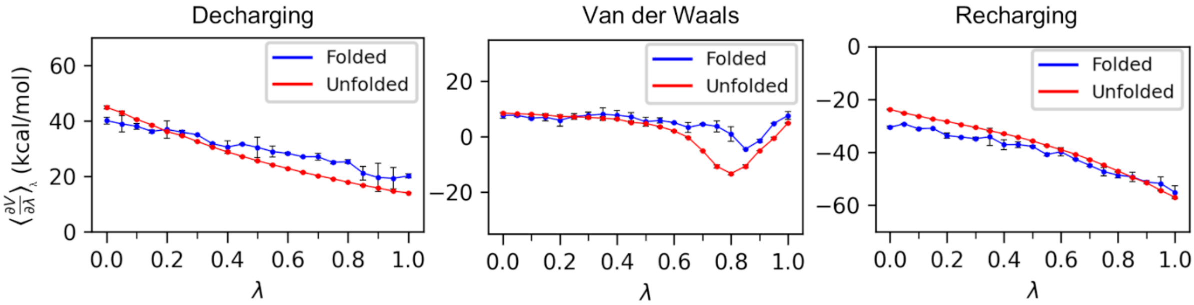


**Figure S17. Average partial derivative of the potential energy with respect to the coupling parameter $\langle \partial V/\partial \lambda \rangle_\lambda$ as a function of λ during alchemical transformations performed to assess the energetic impact of the W3S mutation on Cx32 folding.** Each panel represents a distinct alchemical process, as indicated above each graph, for both systems: the folded state (blue) and the unfolded state (red). The folded state corresponds to the hemichannel in its native conformation, whereas the unfolded state refers to a linear, capped tripeptide (ACE-Gly-X-Gly-NME), where X is either W or S. Error bars represent the standard error of the mean calculated from independent simulations at each λ value.

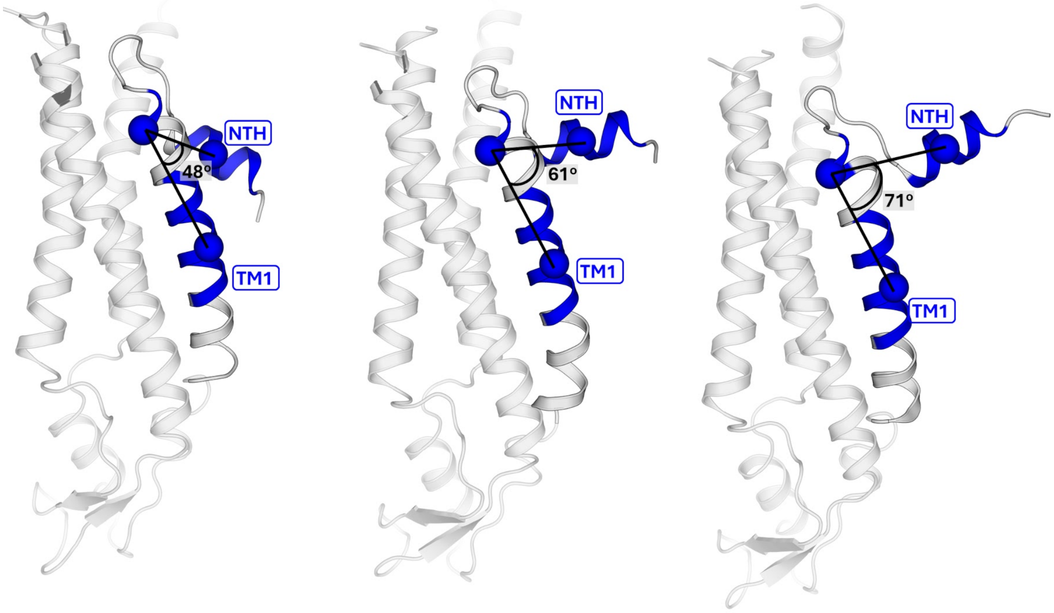


**Figure S18. Definition of the angle used to monitor NTH motion relative to TM1.** The centers of mass (blue spheres) of three groups of Cα atoms, corresponding to residues 3–10, 19–21, and 26–35 in each chain (represented by blue segments), were used to define the NTH-TM1 angle. In the cryo-EM structure of Cx32 in nanodiscs, this angle is approximately 61° (center structure). During MD simulations, both smaller and larger angles are sampled (see left and right structures).

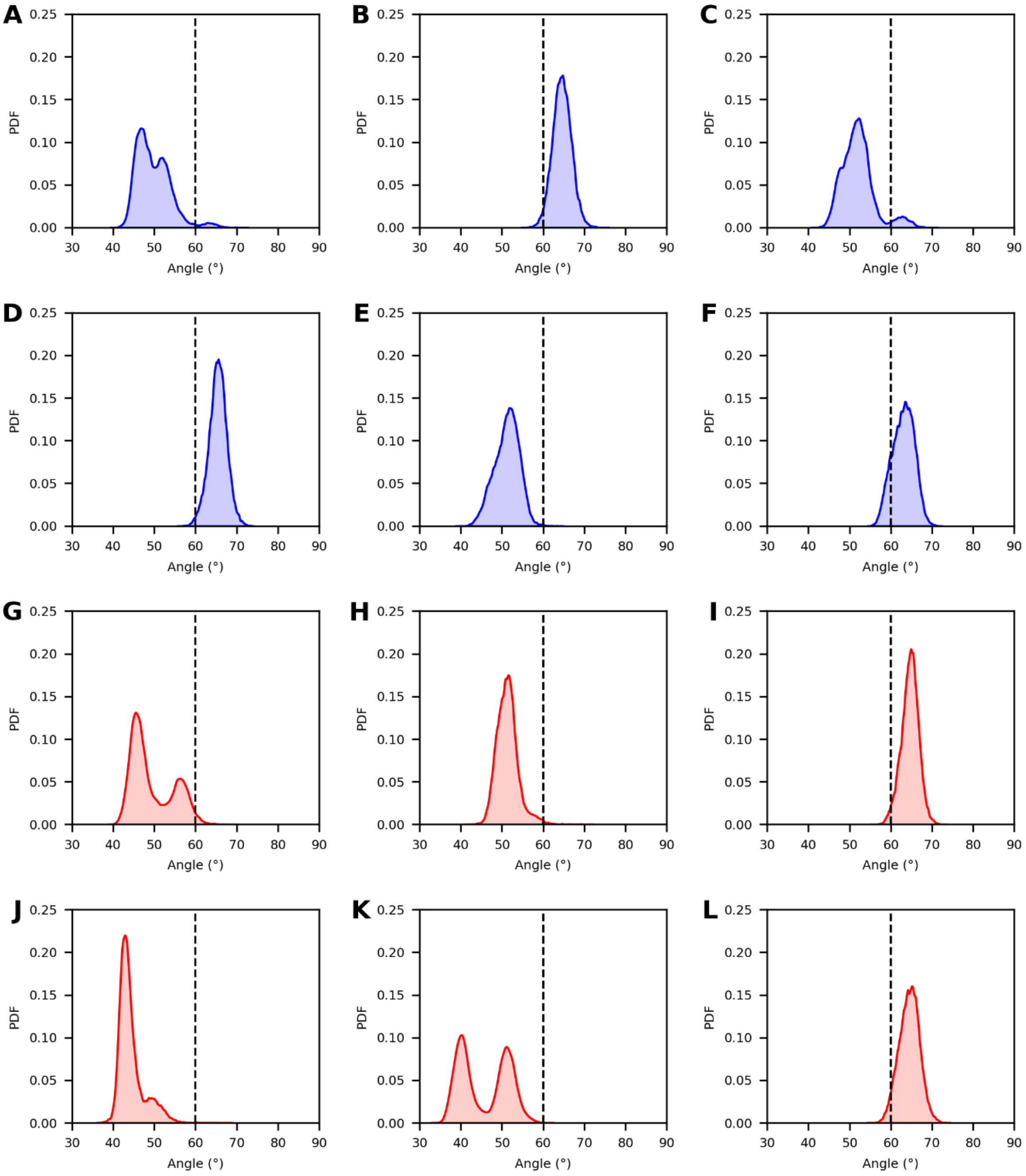


**Figure S19.** Distributions of NTH-TM1 angles in Cx32 wt. **(A–F)** Angle distributions for each chain of the Cx32 hexamer during the first replicate MD simulation (n=6, blue). **(G–L)** Angle distributions for each chain during the second replicate MD simulation (n=6, red). Initial and final panels (A, F and G, L), and adjacent panels represent neighbouring chains in the structure. The vertical dashed line indicates the value of the angle in the cryo-EM structure of Cx32 in nanodiscs.

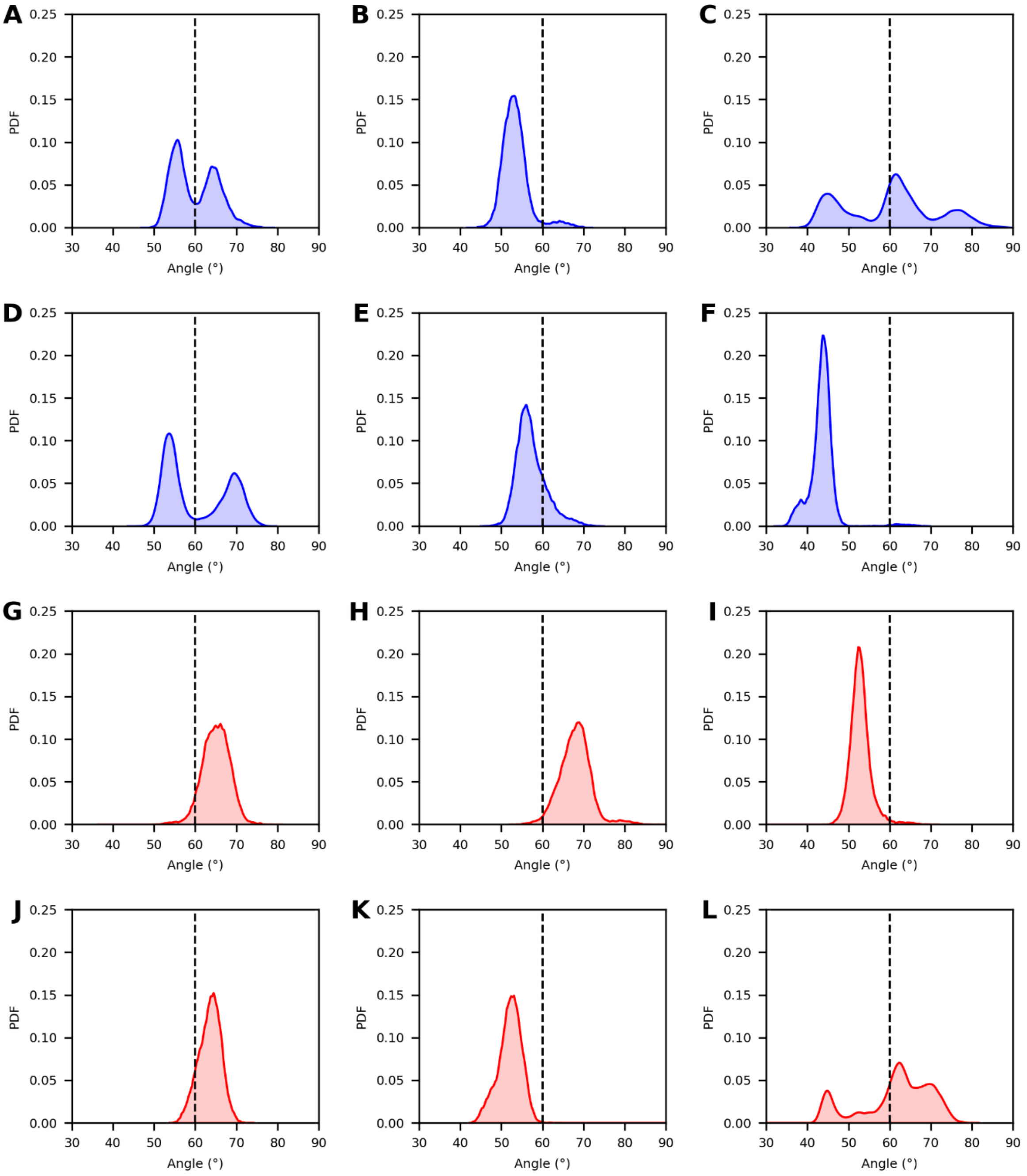


**Figure S20.** Distributions of NTH-TM1 angles in Cx32 W3S. **(A–F)** Angle distributions for each chain of the Cx32 hexamer during the first replicate MD simulation (n=6, blue). **(G–L)** Angle distributions for each chain during the second replicate MD simulation (n=6, red). Initial and final panels (A, F and G, L), and adjacent panels represent neighbouring chains in the structure. The vertical dashed line indicates the value of the angle in the cryo-EM structure of Cx32 in nanodiscs.

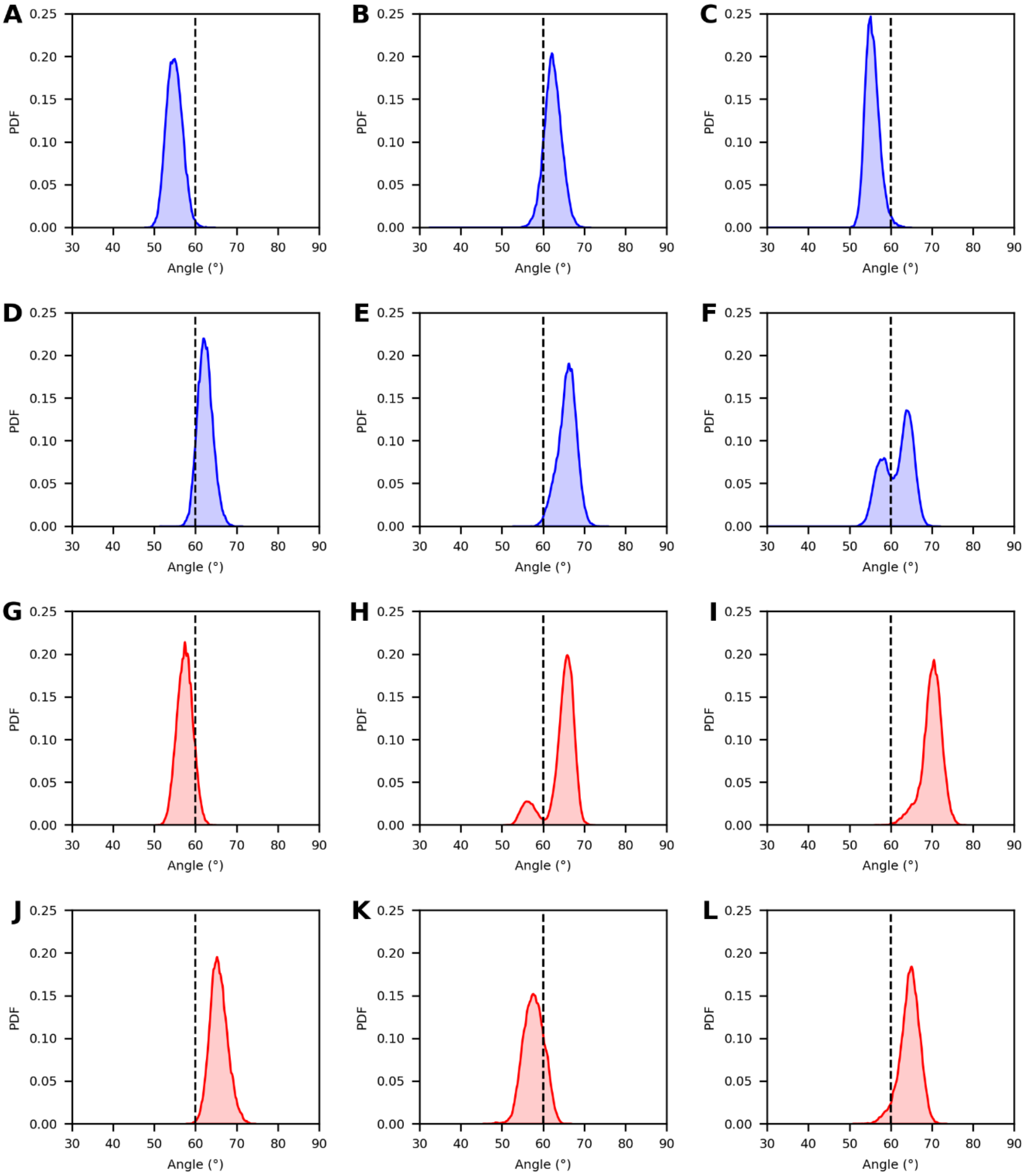


**Figure S21.** Distributions of NTH-TM1 angles in Cx32 wt:POPC:CHOL. **(A–F)** Angle distributions for each chain of the Cx32 hexamer during the first replicate MD simulation (n=6, blue). **(G–L)** Angle distributions for each chain during the second replicate MD simulation (n-6, red). Initial and final panels (A, F and G, L), and adjacent panels represent neighbouring chains in the structure. The vertical dashed line indicates the value of the angle in the cryo-EM structure of Cx32 in nanodiscs.

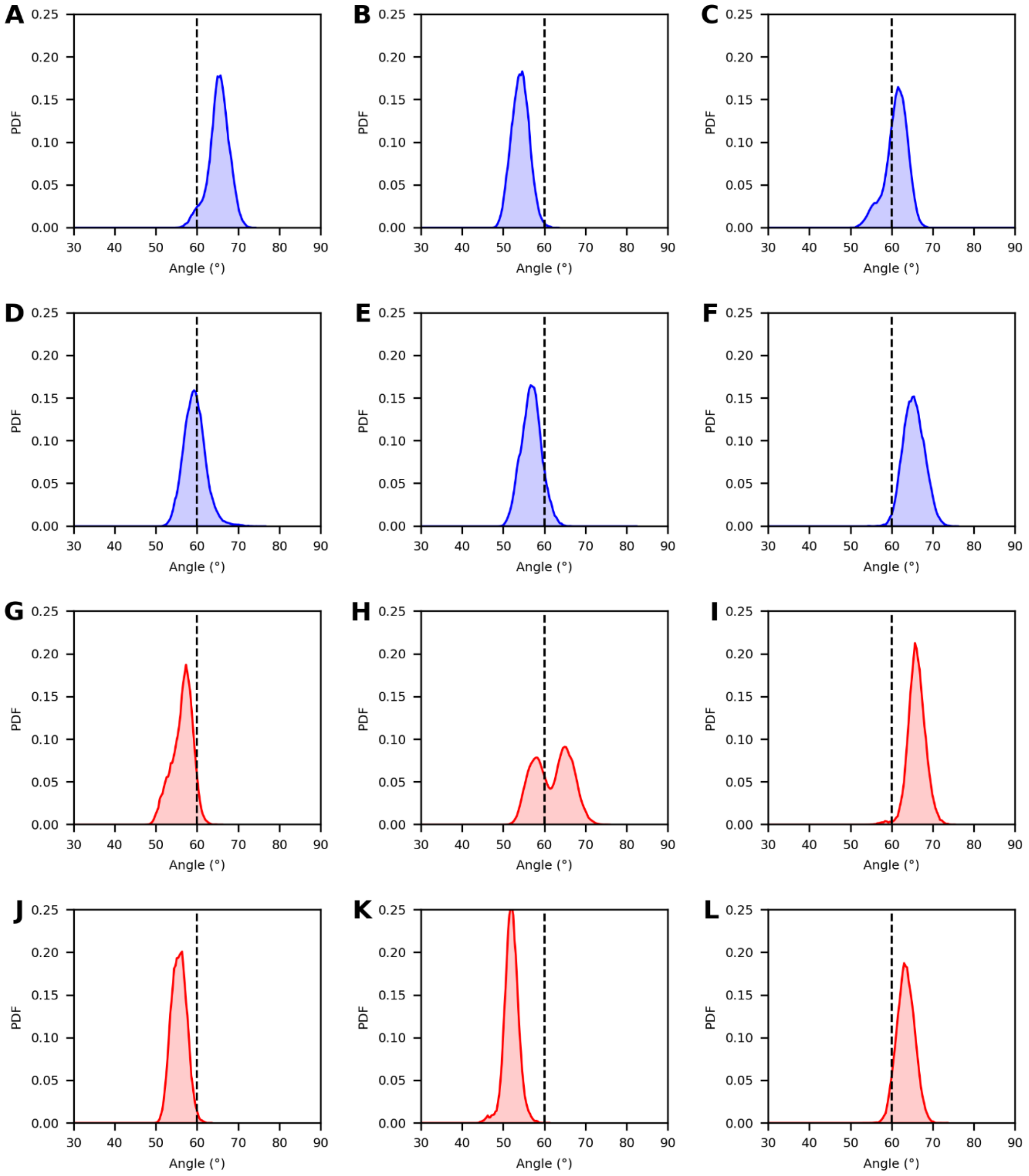


**Figure S22.** Distributions of NTH-TM1 angles in Cx32 W3S:POPC:CHOL. **(A–F)** Angle distributions for each chain of the Cx32 hexamer during the first replicate MD simulation (n=6, blue). **(G–L)** Angle distributions for each chain during the second replicate MD simulation (n=6, red). Initial and final panels (A, F and G, L), and adjacent panels represent neighbouring chains in the structure. The vertical dashed line indicates the value of the angle in the cryo-EM structure of Cx32 in nanodiscs.

## Supplementary Tables

**Table S1. Statistical comparison of the mean probabilities of NTH residues adopting an α-helical conformation in different wild-type and W3S Cx32 systems**

| Systems | $p_{\alpha H}$[a] (Mean ± SEM) | Normality (Shapiro-Wilk, p) | Variance Equality (Levene's Test, p) | Test Used (statistic(df), p)[e] | Effect Size (Cohen's d / Biserial rank r)[f] | 95% CI Mean Diff[g] | Summary |
|---|---|---|---|---|---|---|---|
| Cx32 WT<br>Cx32 W3S | 0.765 ± 0.041<br>0.568 ± 0.076 | 0.901, 0.162<br>0.910, 0.215 | 3.647, 0.069 | Student's t-test:<br>t(22)=2.217, p=0.037(*) [b] | d=0.905 | (0.037, 0.372) | Normal distributions; equal variances; significant difference by Student's t-test |
| Cx32 WT:POPC:CHOL<br>Cx32 W3S:POPC:CHOL | 0.807 ± 0.051<br>0.690 ± 0.058 | 0.827, 0.019(*)[c]<br>0.892, 0.127 | - | Mann-Whitney U test:<br>U=94.000, p=0.214 | r=0.306 | | One group not normal; no significant difference by Mann-Whitney U test |
| Cx32 WT<br>Cx32 WT:POPC:CHOL | 0.765 ± 0.041<br>0.807 ± 0.051 | 0.901, 0.162<br>0.827, 0.019(*) | - | Mann-Whitney U test:<br>U=61.000, p=0.544 | r=-0.153 | - | One group not normal; no significant difference by Mann-Whitney U test |
| Cx32 W3S<br>Cx32 W3S:POPC:CHOL | 0.568 ± 0.076<br>0.690 ± 0.058 | 0.910, 0.215<br>0.892, 0.127 | 0.730, 0.402 | Student's t-test:<br>t(22)=-1.272, p=0.402 | d=-0.519 | (-0.310,0.066) | Normal distributions; equal variances; no significant difference by Student's t-test |
| Cx32 WT:CHOL<br>Cx32 W3S:CHOL | 0.834 ± 0.022<br>0.689 ± 0.058 | 0.917, 0.264<br>0.820, 0.016(*) | - | Mann-Whitney U test:<br>U=103.000, p=0.078 | r=0.431 | - | One group not normal; no significant difference by Mann-Whitney U test |
| Cx32 WT<br>Cx32 WT:CHOL | 0.765 ± 0.041<br>0.834 ± 0.022 | 0.901, 0.162<br>0.917, 0.264 | 1.597, 0.192 | Student's t-test:<br>t(22)=-1.345, p=0.192 | d=-0.549 | (-0.169, 0.031) | Normal distributions; equal variances; no significant difference by Student's t-test |
| Cx32 W3S<br>Cx32 W3S:CHOL | 0.568 ± 0.076<br>0.689 ± 0.058 | 0.910, 0.215<br>0.820, 0.016(*) | - | Mann-Whitney U test:<br>U=55.000, p=0.341 | r=-0.236 | - | One group not normal; no significant difference by Mann-Whitney U test |
| Cx32 WT:POPC<br>Cx32 W3S:POPC | 0.847 ± 0.023<br>0.666 ± 0.062 | 0.949, 0.617<br>0.928, 0.361 | 15.141,<br>0.001(***)[d] | Welch's t-test:<br>t(14.0)=2.763, p=0.015(*) | d=1.128 | (0.053,0.310) | Normal distribution; unequal variances; significant difference by Welch's t-test |
| Cx32 WT<br>Cx32 WT:POPC | 0.765 ± 0.041<br>0.847 ± 0.023 | 0.901, 0.162<br>0.847 ± 0.023 | 1.34, 0.259 | Student's t-test:<br>t(22)=-1.598, p=0.122 | d=-0.653 | (-0.182, 0.019) | Normal distributions; equal variances; no significant difference by Student's t-test |
| Cx32 W3S<br>Cx32 W3S:POPC | 0.568 ± 0.076<br>0.666 ± 0.062 | 0.910, 0.215<br>0.928, 0.361 | 0.300, 0.589 | Student's t-test:<br>t(22)=-0.998, p=0.329 | d=-0.408 | (-0.290,0.094) | Normal distributions; equal variances; no significant difference by Student's t-test |

[a] Probability of residues 3–5 and 9–10 adopting an α-helix conformation during the last 400 ns of the replicate MD simulations conducted for each system· ·Mean values and standard errors of the mean (SEM) are reported for n = 12 measurements per system (6 monomers × 2 replicate simulations; monomers were initialized with independent velocities and are treated as independent samples).

[b] An asterisk indicates a statistically significant difference between the compared mean values ($p \leq 0.05$).

[c] An asterisk indicates a significant deviation from normality ($p \leq 0.05$).

[d] Three asterisks indicate that the standard deviations are significantly different ($p \leq 0.001$).

[e] Statistical test used for each pairwise comparison. Reported values correspond to the test statistic (and degrees of freedom, when applicable) followed by the exact p-value. For non-parametric tests (e.g., Mann–Whitney U), degrees of freedom are not applicable and therefore omitted. All tests were two-tailed.

[f] Effect sizes were calculated as follows: for parametric tests, Cohen's *d* was computed as the difference between group means divided by the pooled standard deviation ($d = (M_1 - M_2)/SD_{pooled}$); for non-parametric tests, the rank-biserial correlation (*r*) was derived from the Mann–Whitney U statistic ($r = 1 - 2U/[n_1 \times n_2]$) and its sign was adjusted so that *r* is positive when the mean of group 1 exceeds that of group 2.

[g] 95% confidence interval (CI) for the mean difference between the compared groups, expressed as (lower bound, upper bound). The interval reflects the range within which the true mean difference is expected to lie with 95% confidence. Confidence intervals are reported only for parametric tests (Student's *t*-test and Welch's *t*-test).

**Table S2.** Alchemical ΔΔG value associated with the W3S mutation in the Cx32 folded state and the unfolded state

| | Unfolded | Folded | Unfolded – Folded |
|---|---|---|---|
| **$\Delta G_{dch}$ (kcal/mol)[a]** | 27.02 (0.03) | 29.87 (0.34) | -2.85 (0.34) |
| **$\Delta G_{vdw}$ (kcal/mol)[b]** | 1.84 (0.06) | 5.10 (0.22) | -3.26 (0.22) |
| **$\Delta G_{rch}$ (kcal/mol)[c]** | -37.46 (0.03) | -39.95 (0.21) | 2.49 (0.21) |
| **ΔΔG (kcal/mol)** | - | - | -3.62 (0.46) |

[a]Free energy associated with the W3 decharging process.
[b]Free energy associated with the W3→S3 van der Waals transformation
[c]Free energy associated with the S3 recharging process.

**Table S3.** Description of composition and simulation boxes for systems prepared for TI free energy calculations

<table>
<tr><th>System[a]</th><th>No. of Counterions</th><th>No. of water molecules</th><th>No. of atoms</th><th>Initial box dimensions (Å)</th></tr>
<tr><td>Cx32 WT (decharging)</td><td rowspan="3">36 $Cl^-$</td><td rowspan="3">30,971</td><td>112,041</td><td rowspan="3">x: 109.348<br>y:118.413<br>z: 102.342</td></tr>
<tr><td>Cx32 W3S (recharging)</td><td>112,021</td></tr>
<tr><td>Cx32 W3/S3 (van der Waals)</td><td>112,028</td></tr>
<tr><td>GWG (decharging)</td><td rowspan="3">0</td><td>1,668</td><td>5,078</td><td rowspan="3">x: 40.217<br>y: 46.128<br>z: 37.797</td></tr>
<tr><td>GSG (recharging)</td><td>1,513</td><td>4,587</td></tr>
<tr><td>G(W/S)G (van der Waals)</td><td>1,668</td><td>5,065</td></tr>
</table>

[a]Different systems were prepared for each alchemical transformation (decharging, recharging and van der Waals transformations), comprising the protein (Cx32 WT or W3S) and the capped tripeptide (GXG, X=S/W).
