# Supplementary material for: Lipid dependence of connexin-32 gap junction channel conformations
Source: Nat Commun. 2025 Dec 5;17:316. doi: 10.1038/s41467-025-67004-z (PMC12789061; doi:10.1038/s41467-025-67004-z)
Supplement: Supplementary file 2 — Reporting Summary [file 41467_2025_67004_MOESM2_ESM.pdf]

nature portfolio

Corresponding author(s): V. Korkhov

Last updated by author(s): Nov 9, 2025

# Reporting Summary

Nature Portfolio wishes to improve the reproducibility of the work that we publish. This form provides structure for consistency and transparency in reporting. For further information on Nature Portfolio policies, see our Editorial Policies and the Editorial Policy Checklist.

## Statistics

For all statistical analyses, confirm that the following items are present in the figure legend, table legend, main text, or Methods section.

| n/a | Confirmed | |
|---|---|---|
| ☐ | ☒ | The exact sample size ($n$) for each experimental group/condition, given as a discrete number and unit of measurement |
| ☐ | ☒ | A statement on whether measurements were taken from distinct samples or whether the same sample was measured repeatedly |
| ☐ | ☒ | The statistical test(s) used AND whether they are one- or two-sided<br>*Only common tests should be described solely by name; describe more complex techniques in the Methods section.* |
| ☒ | ☐ | A description of all covariates tested |
| ☐ | ☒ | A description of any assumptions or corrections, such as tests of normality and adjustment for multiple comparisons |
| ☐ | ☒ | A full description of the statistical parameters including central tendency (e.g. means) or other basic estimates (e.g. regression coefficient) AND variation (e.g. standard deviation) or associated estimates of uncertainty (e.g. confidence intervals) |
| ☐ | ☒ | For null hypothesis testing, the test statistic (e.g. $F$, $t$, $r$) with confidence intervals, effect sizes, degrees of freedom and $P$ value noted<br>*Give P values as exact values whenever suitable.* |
| ☒ | ☐ | For Bayesian analysis, information on the choice of priors and Markov chain Monte Carlo settings |
| ☒ | ☐ | For hierarchical and complex designs, identification of the appropriate level for tests and full reporting of outcomes |
| ☐ | ☒ | Estimates of effect sizes (e.g. Cohen's $d$, Pearson's $r$), indicating how they were calculated |

*Our web collection on statistics for biologists contains articles on many of the points above.*

## Software and code

Policy information about availability of computer code

| | |
|---|---|
| Data collection | EPU (Thermo Fischer) - software for cryo-EM data collection |
| Data analysis | Relion 4.0.1, MotionCor2, Ctffind4, Gctf, Coot |

For manuscripts utilizing custom algorithms or software that are central to the research but not yet described in published literature, software must be made available to editors and reviewers. We strongly encourage code deposition in a community repository (e.g. GitHub). See the Nature Portfolio guidelines for submitting code & software for further information.

## Data

Policy information about availability of data

All manuscripts must include a data availability statement. This statement should provide the following information, where applicable:

- Accession codes, unique identifiers, or web links for publicly available datasets
- A description of any restrictions on data availability
- For clinical datasets or third party data, please ensure that the statement adheres to our policy

The MD simulation trajectories and data have been deposited to the OSF repository (https://osf.io/gezpx/). All coordinates and cryo-EM density maps have been deposited to the Protein Data Bank (9QN9, 9QND, 9QNF, 9QNT) and Electron Microscopy Data Bank (EMD-53240, EMD-53244, EMD-53245, EMD-53250).

## Research involving human participants, their data, or biological material

Policy information about studies with human participants or human data. See also policy information about sex, gender (identity/presentation), and sexual orientation and race, ethnicity and racism.

| | |
|---|---|
| Reporting on sex and gender | N/A |
| Reporting on race, ethnicity, or other socially relevant groupings | N/A |
| Population characteristics | N/A |
| Recruitment | N/A |
| Ethics oversight | N/A |

Note that full information on the approval of the study protocol must also be provided in the manuscript.

# Field-specific reporting

Please select the one below that is the best fit for your research. If you are not sure, read the appropriate sections before making your selection.

[x] Life sciences [ ] Behavioural & social sciences [ ] Ecological, evolutionary & environmental sciences

For a reference copy of the document with all sections, see nature.com/documents/nr-reporting-summary-flat.pdf

# Life sciences study design

All studies must disclose on these points even when the disclosure is negative.

| | |
|---|---|
| Sample size | No statistical methods were used to define the sample size. The work focused on cryo-EM analysis of several defined protein complexes. |
| Data exclusions | The standard 2D and 3D classification protocols were used to classify the particles during cryo-EM analysis. Otherwise, no data exclusion was performed. |
| Replication | To ensure that the structural observations with a chemically defined lipid (POPC) were reproducible, we also performed a similar analysis with a natural lipid extract (liver polar lipids, LPL). The results showed a similar pattern, confirming that the observations are reproducible. The work focused on POPC-containing sample. |
| Randomization | Randomization was not relevant to our study, as we had only a limited set of parameters to vary: we used only two protein constructs (Cx32 wild-type and Cx32-W3S mutant), two lipid types (POPC and LPL), one sterol analogue (CHS). While randomization could potentially be performed still, we opted not to do it at the planning stage. |
| Blinding | Blinding could potentially be performed, however we opted to not do it due to a small number of samples analysed. |

# Reporting for specific materials, systems and methods

We require information from authors about some types of materials, experimental systems and methods used in many studies. Here, indicate whether each material, system or method listed is relevant to your study. If you are not sure if a list item applies to your research, read the appropriate section before selecting a response.

### Materials & experimental systems

| n/a | Involved in the study | |
|---|---|---|
| x | | Antibodies |
| | x | Eukaryotic cell lines |
| x | | Palaeontology and archaeology |
| x | | Animals and other organisms |
| x | | Clinical data |
| x | | Dual use research of concern |
| x | | Plants |

### Methods

| n/a | Involved in the study | |
|---|---|---|
| x | | ChIP-seq |
| x | | Flow cytometry |
| x | | MRI-based neuroimaging |

## Eukaryotic cell lines

Policy information about cell lines and Sex and Gender in Research

| | |
|---|---|
| Cell line source(s) | HEK293F - Thermo Fischer Scientific |

| | |
|---|---|
| Authentication | Cell line was not authenticated, but was purchase from a commercial vendor |
| Mycoplasma contamination | Mycoplasma tests were negative |
| Commonly misidentified lines (See ICLAC register) | N/A |

## Plants

| | |
|---|---|
| Seed stocks | N/A |
| Novel plant genotypes | N/A |
| Authentication | N/A |
